# COVID-19 Lockdown and Its Adverse Impact on Psychological Health in Breast Cancer

**DOI:** 10.3389/fpsyg.2020.02033

**Published:** 2020-08-24

**Authors:** Jessica Swainston, Bethany Chapman, Elizabeth A. Grunfeld, Nazanin Derakshan

**Affiliations:** Department of Psychological Sciences, Birkbeck, University of London, London, United Kingdom

**Keywords:** breast cancer, COVID-19, anxiety, depression, psycho-oncology, cognition

## Abstract

The Coronavirus disease 2019 (COVID-19) outbreak generated an unprecedented set of emotional challenges for women diagnosed with breast cancer. In the United Kingdom (UK), the pandemic significantly disrupted oncology services as resources were reassigned to care for COVID-19 patients. In addition to service disruptions, many women received a UK Government letter advising them to shield for 12-weeks. We aimed to explore the effect of disruption to scheduled oncology services and the UK Government shielding letter on emotional and cognitive vulnerability. A further aim was to investigate the relationship between COVID-19 related emotional vulnerability (COVID-EMV) and anxiety, depression and perceived cognitive function. Women diagnosed with primary breast cancer (*N* = 234) completed a series of online questionnaires to assess their cognitive and emotional wellbeing as well as their COVID-EMV. Results indicated that disrupted oncology services had a significant impact on COVID-EMV, anxiety and depression, with those experiencing disruptions expressing higher general emotional vulnerability as well as COVID-EMV. Further, the UK Government letter had a significant effect on perceived cognitive function; those who received the letter reported poorer cognitive function. Regression analyses revealed that after allowing for the effects of sociodemographic and clinical variables, women’s COVID-EMV significantly predicted worse outcomes of anxiety, depression and perceived cognitive function. Our findings indicate that concerns about COVID-19 amongst women affected by breast cancer leads to increased risk of developing affective disorder, such as anxiety and depression symptomatology, among this sample. We advocate the rapid implementation of accessible interventions designed to promote emotional resilience in the breast cancer population.

## Introduction

The coronavirus disease 2019 (COVID-19) outbreak, caused by severe acute respiratory syndrome coronavirus 2 (SARS-CoV-2), has escalated into a global pandemic at a formidable rate. Whilst this has caused unprecedented disruption to populations globally, it is a particularly worrying time for vulnerable groups with pre-existing health conditions, including women with a breast cancer diagnosis. The immunosuppressant effects of cancer and its treatment (Verma et al., 2016), coupled with the multimorbidity that often occurs in cancer patients (Renzi et al., 2019), suggests that those affected by breast cancer and other cancers may be at particular risk from the impact of COVID-19 (Zhang et al., 2020). Indeed, in the UK, recent estimates predict approximately 18,000 excess cancer deaths over the next 12 months as a result of the COVID-19 emergency (Lai et al., 2020).

Additional cancer deaths may result from the direct effects of contracting COVID-19, or the indirect effects of the pandemic on disruption of cancer treatment services and delays in diagnosis. Observations indicate that at the height of the emergency, across eight hospitals in the UK, a majority of patients with cancer or suspected cancer were not accessing healthcare services, with major declines in chemotherapy attendances (60% reduction) and urgent referrals for early diagnosis (76% reduction) (Lai et al., 2020). Early diagnosis and intervention are critical factors for survival outcomes in breast cancer. Recent data indicates that 100% of women diagnosed at Stage 1 survive their disease for at least one year, compared to 66% diagnosed at Stage 4 (Office for National Statistics, 2019). However, in the midst of a global pandemic, it is understandable that individuals with ambiguous symptoms, which may or may not reflect cancer disease, are reluctant to seek help.

In addition to delays in breast cancer diagnosis, and disruption of active treatment plans (e.g., chemotherapy, radiotherapy, surgery), women in survivorship may also experience adverse effects from the current pandemic. Once active treatment is complete, many breast cancer survivors are still expected to attend routine checkups, have regular scans, or yearly mammograms, many of which have been disrupted as a consequence of COVID-19. For those with estrogen-positive breast cancer, treatment can extend to 10 years post-diagnosis, with the administration of endocrine therapies, such as tamoxifen or aromatase inhibitors, to reduce the risk of recurrence. As such, continued access to healthcare services is important to women in the breast cancer survivorship period.

The debilitating physical and psychological side effects of breast cancer diagnosis, both during and after treatment, are well documented (Härtl et al., 2010; Hagen et al., 2016). Whilst studies indicate that emotional distress is at its most severe during diagnosis and active treatment (Schubart et al., 2014), clinically important distress can persist in survivorship (Carreira et al., 2018). Long term sequela including fatigue, sleep disturbance, joint pain and hot flushes, as well as sexual dysfunction, can profoundly affect quality of life (Fallowfield and Jenkins, 2015). For younger women, further stress may derive from the potential onset of early menopause and loss of fertility caused by estrogen depleting endocrine therapies (Gorman et al., 2010). Negative thinking patterns are also common including ruminative health-related thinking styles and concerns related to the fear of cancer recurrence and one’s own mortality (Steiner et al., 2014; Thewes et al., 2016). Neurocognitive dysfunction is a further common problem among the breast cancer population (see Andryszak et al., 2017; Ahles and Root, 2018 for reviews) and may be associated with adverse mental health outcomes (Chapman et al., 2019). Accordingly, the breast cancer population are at high-risk for developing clinical levels of emotional distress, which can result in affective disorders such as anxiety, depression and post-traumatic stress disorder (PTSD; Steiner et al., 2014; Voigt et al., 2017; Tsaras et al., 2018).

Given the unsettling nature of the COVID-19 crisis, emotional distress symptomatology in the breast cancer population may be exacerbated. Along with treatment disruptions, many women living in the UK affected by breast cancer will have received a UK Government letter categorizing them as vulnerable and outlining a 12-week social restriction and shielding plan (Gov.UK, 2020). Recipients of the letter may include those undergoing chemotherapy, immunotherapy or having other targeted cancer treatments that can affect the immune system, such as protein kinase inhibitors or PARP inhibitors. Others may have received a letter at the discretion of their general practitioner (GP). Such guidelines are also likely to increase social isolation and loneliness (Holmes et al., 2020), which are independently associated with anxiety, depression, and self-harm (Elovainio et al., 2017). It is currently unclear how long vulnerable populations will be affected by the impact of the COVID-19 crisis; however, the ramifications may be severe. It follows that understanding the impact of the COVID-19 outbreak on cognitive and emotional health is critical to supporting the breast cancer population moving forward.

With the above concerns in mind, the current investigation was two-fold. First, we decided to investigate the effect of disruption to scheduled oncology services (i.e., delayed treatment or canceled scans) and the UK Government shielding letter on emotional vulnerability and perceived cognitive function in a group of women affected by primary breast cancer. We then decided to examine the relationship between COVID-19 related emotional vulnerability (COVID-EMV) and general anxiety, general depression and perceived cognitive function whilst controlling for rumination, worry, and key clinical and sociodemographic variables. Worry, which is characterized by uncontrollable affectively negative thoughts about the future (Borkovec et al., 1983), and rumination, distinguished by repetitive affectively negative thoughts (Nolen-Hoeksema et al., 2008), are core cognitive components of clinical anxiety and depression respectively (Beckwé et al., 2014). As such, trait ruminative and worrisome thinking styles are key predictors for emotional disorder (Nolen-Hoeksema, 2000; see Koster et al., 2017 for a review). Indeed the bidirectional relationship between cognition and emotion is well established (Pessoa, 2008), with cognitive health playing an important role in emotional regulation and vice versa (Dolcos et al., 2019). With this in mind, we felt that it was important to allow for the predictive value of worry and rumination whilst assessing the impact of COVID-EMV on anxiety, depression and perceived cognitive function. The additive stress caused by the COVID-19 outbreak, which may compound worrisome and ruminative thinking patterns, may predict worse outcomes for anxiety, depression and cognitive health in women affected by breast cancer, who already consistently indicate cognitive and emotional vulnerability. Accordingly, we predicted that compared to those unaffected, women who experienced changes to their scheduled oncology services or appointments (i.e., telephone appointments in place of face-to-face) or who received the UK Government shielding letter would express higher levels of general emotional vulnerability (i.e., anxiety and depression) and COVID-EMV as well as a worse perceived cognitive function. In addition, we predicted that worry and rumination would respectively predict anxiety and depression as well as perceived cognitive function. Finally, we predicted that after controlling for worry, rumination and key clinical and sociodemographic variables, COVID-EMV would significantly predict worse outcomes for general anxiety, general depression and perceived cognitive function.

## Materials and Methods

### Design

The design was cross-sectional. Participants were asked to complete a series of online questionnaires measuring cognitive and emotional health and the specific impact of the COVID-19 outbreak restrictions on emotional vulnerability.

### Participants

Women with a diagnosis of primary breast cancer were recruited through voluntary sampling using advertisements placed on support platforms including, “Building Resilience in Breast Cancer Centre” (BRiC Centre)^1^. Participants could be at any stage of diagnosis and treatment (i.e., about to start treatment, receiving active treatment or post-treatment).

### Materials

#### Demographic and Clinical Questionnaire

The Demographic and Clinical Questionnaire (DQ) was developed by the researchers to collect information regarding the participant’s breast cancer history, sociodemographic characteristics, and history of psychological conditions (i.e., anxiety or depression). All information was self-reported by the participant.

#### Functional Assessment of Cancer Therapy-Cognitive Scale (Version 3)

The Functional Assessment of Cancer Therapy-Cognitive Scale (FACT-Cog, Version 3; Wagner et al., 2009) is a highly reliable self-report inventory composed of 37-items that measure both perceived cognitive impairments (i.e., “My thinking has been slow”) and perceived cognitive ability (i.e., “I have been able to concentrate”) over the last seven days. The Fact-Cog also assesses the impact of these cognitive changes on quality of life and the comments that have been made by others (i.e., “other people have told me I seemed confused”). Questions are measured on five-point Likert scale ranging from not at all or never (0) to several times a day or very much (5), with a total score ranging from 0 to 148. Higher scores indicate better cognitive function and quality of life. The Fact-Cog has been widely implemented in previous breast cancer research (Von Ah and Tallman, 2015; Janelsins et al., 2017; Vardy et al., 2019). Analysis showed an excellent reliability (Cronbach’s α = 0.97) in the present study.

#### Rumination Response Scale

The Rumination Response Scale (RRS; Treynor et al., 2003) is a 22-item self-report questionnaire that measures the level of depressive rumination experienced. Items are measured on a four-point Likert scale ranging from 1 (never) to 4 (almost always), with scores ranging from 22 to 88. A higher total score represents a greater level of rumination. The RRS is a highly reliable questionnaire which has previously been used in breast cancer studies (Swainston and Derakshan, 2018). An excellent Cronbach’s α was found in the current study (Cronbach’s α = 0.94).

#### Hospital Anxiety and Depression Scale

The self-report Hospital Anxiety and Depression Scale (HADS; Zigmond and Snaith, 1983) is a widely used scale that measures anxiety and depression experienced over the last seven days (Osborne et al., 2004; Gregorowitsch et al., 2019). The HADS consists of 14-items, seven relating to anxiety symptomatology and seven relating to depression symptomatology. All items are measured on a four-point Likert scale from 0 to 3, with total scores ranging from 0 to 21 for each of the subsections. Higher scores indicating greater levels of anxiety and/or depression. A high reliability was found in the present study (Cronbach’s α = 0.89).

#### Penn State Worry Questionnaire

The Penn State Worry Questionnaire (PSWQ) (Meyer et al., 1990) is a self-report questionnaire composed of 16 positively or negatively phrased items that measure trait worry. Items are measured on a five-point Likert scale ranging from 1 (“not typical of me”) to 5 (“very typical of me”), with higher scores representing a greater level of pathological worry (PSWQ total score ranges from 16 to 80). The PSWQ is a reliable and valid questionnaire which has been used in breast cancer research (Shapiro et al., 2003; Swainston and Derakshan, 2018). An excellent Cronbach’s α was found in the present study (Cronbach’s α = 0.94).

#### Modified Self-Report-Generated Charlson Comorbidity

The modified Charlson Comorbidity Index (CCI; Charlson et al., 1987) is composed of nine comorbidity items that identify health conditions (i.e., diabetes) experienced by an individual at the present time. Items are each given a weighted value (1, 2, 3, or 6) and the CCI total score is calculated from the summation of these values (0–19). A higher score indicates more chronic comorbidity. The CCI has been used to measure comorbidity in women diagnosed with breast cancer (Ording et al., 2013; Fu et al., 2015). Reliability analysis showed a Cronbach’s α (0.13).

#### COVID-19 Impact Questions

Twenty-four items were developed by the authors, whose expertise lie in psycho-oncology and affective neuroscience, to explore the impact of the COVID-19 outbreak and the restrictive measures on women with a diagnosis of breast cancer. Five items assessing the impact of the outbreak on emotional wellbeing (i.e., “Has the COVID-19 outbreak made you feel more: (1) anxious, (2) upset, (3) fearful than usual” or “Has the COVID-19 outbreak made you feel less: (4) in control, or (5) less confident than usual”) were included, with higher scores indicating a higher level of COVID-19 generated emotional vulnerability. A composite score derived from the five emotional vulnerability items was formed and reliability analysis showed a Cronbach’s α = 0.89^2^ (see Supplementary Material for item reliability, factor analysis and COVID-EMV correlations). In addition, individual items assessing isolation status (i.e., yes or no), the UK Government shielding letter (i.e., yes or no) and COVID-19 disruption to scheduled appointments (i.e., yes or no) were included. These individual items were utilized in our study to explore the effect of COVID-19 related disruption and restrictive measures (i.e., shielding letter) on women’s emotional health (anxiety and depression).

Personal experience of COVID-19 symptoms (high temperature/fever, continuous cough, shortness of breath, chest pain or pressure, sore throat, sneezing or runny nose, loss of smell and/or taste, bluish lips or face or new confusion, or inability to arouse)^3^ was also independently measured. This allowed us to examine whether COVID related symptoms had any impact on our relationships of interest^4^.

### Procedure

After registering an interest in the study, participants were emailed an information document outlining the primary aim of the research as well as a secure URL code to access the battery of self-report questionnaires. Participants completed an online consent form before being directed onto the DQ. This was followed by the cognitive and emotional wellbeing questionnaires as well as the main COVID-19 questionnaire. Upon completion of the study, participants received a £5 gift voucher. This research received ethical approval from the Research Ethics Committee of the Department of Psychological Sciences and the College Research Ethics Committee at Birkbeck College, University of London as well as the Economic and Social Research Council.

### Statistical Analysis

Statistical analyses were performed using the Statistical Package for the Social Sciences (SPSS, version 25). Descriptive statistics were produced for the sociodemographic characteristics, clinical history of breast cancer and history of psychological disorders (see Table 1). A series of 2 × 2 ANOVAs were performed to examine the main effects of the UK Government shielding letter and disruption to scheduled oncology services and the interaction effect between the UK Government shielding letter and disruption to oncology services on women’s COVID-19-specific-emotional vulnerability (COVID-EMV) as well as their general anxiety, depression and perceived cognitive function. Three-stage hierarchical regression analyses were performed to investigate the relationships between COVID-EMV and three dependent variables: general anxiety, general depression and perceived cognitive ability after allowing for the effects of a series of sociodemographic and clinical predictors. Model one included six demographic and clinical factors: (1) grade, (2) active treatment status, (3) time since diagnosis, (4) age at diagnosis, (5) education, and (6) health co-morbidities (measured by CCI). Measures of ruminative behavior and pathological worry were then added in model two and COVID-EMV as a final predictor in the last model (model three). Using analysis of standardized residual, no outliers were found (Anxiety: std Residual Min = -2.31, std Residual Max = 2.53, std deviation = 0.98; Depression: std Residual Min = -2.60, std Residual Max = 3.14, std deviation = 0.98; Perceived cognitive function: std Residual Min = -2.73, std Residual Max = 2.57, std deviation = 0.98). Moreover, checks for violations of the assumptions of collinearity, independent error, normality, homoscedasticity and linearity were also performed and all assumptions were met.

**TABLE 1:** Participant demographics, clinical characteristics, and psychiatric history.

	*N* = 234 (%)
**Sociodemographic**	
Age	Mean = 51 Years (Min = 27, Max = 78)
*Education*	
Secondary Education	26 (11.1)
Further Education	50 (21.4)
Higher Education	152 (65.0)
Other	6 (2.6)
*Ethnicity*	
White	222 (94.9)
Black	3 (1.3)
Asian	5 (2.1)
Multi-ethnic	3 (1.3)
*Civil Status*	
Married/Civil Partnership/Cohabiting	173 (73.9)
Divorced/Separated	19 (8.1)
Single/Widowed	38 (16.2)
**Work**	
*Employment status*	
Employed	147 (62.8)
Self-employed	25 (10.7)
Undertaking volunteering work	14 (6.0)
Not undertaking any form of work	48 (20.5)
**Clinical – Breast Cancer History**	
Age at Diagnosis	Mean = 47 Years (Min = 24, Max = 77)
Time Since Diagnosis	Mean = 51.46 Months (Min = 0, Max = 177)
*Grade*	
Grade 1	28 (12.0)
Grade 2	86 (36.8)
Grade 3	117 (50.0)
*Active Treatment*	
Yes	15 (6.4)
No	215 (91.9)
Due to Start	2 (0.9)
Other	2 (0.9)
*Type of Treatment Received*	
Chemotherapy	171 (73.1)
Radiotherapy	186 (79.5)
Surgery	
Mastectomy	97 (41.5)
Lumpectomy	98 (41.9)
Mastectomy and Lumpectomy	23 (9.8)
*Endocrine Therapy*	
Yes	161 (68.8)
No	63 (26.9)
Other (i.e., Prescribed but decided not to take it)	10 (4.3)
Time Since Treatment Finished	Mean = 38 Months (Min = 0, Max = 140)
History of Psychological Disorders	100 (42.7)
Prescribed medication for conditions other than cancer	49 (20.9)

*^a^One participant did not disclose their ethnicity, ^b^Four participants did not disclose their civil status. ^c^Three participants did not state the grade of their breast cancer. ^d^One participant did not disclose the treatment they received.*

## Results

Of the 234 women (*mean age* = 51, *SD* = 7.88; *mean age at diagnosis* = 47, *SD* = 7.68) recruited to participate in the current study, 23% (54 women) had received a UK Government shielding letter. A total of 74 women (31.6%) had been impacted by disruption to their scheduled oncology services (i.e., had appointments canceled or delivered over the phone instead of in person) (see Table 1 for demographics) and 24 (10.3%) had received both the government letter and experienced disruption to their schedule oncology services or appointments. Only 35 from 234 women (15%) reported that they had shown COVID-19 related symptoms. About 10% of symptoms reported were a fever and/or cough. None of these participants reported that they had been clinically diagnosed with the virus.

### Impact of Oncology Service Disruptions and Government Shielding Letter

Four 2 × 2 ANOVA’s were conducted to examine the main effects of the UK Government shielding letter and the impact of disruption to oncology services and the interaction effect between the UK Government shielding letter and disruption to oncology services on women’s COVID-EMV as well as their anxiety, depression and perceived cognitive function. The results showed that disruption to scheduled oncology services had a significant main effect on women’s COVID-EMV (*F* (1, 230) = 9.68, *p* = 0.002), general anxiety (*F* (1, 230) = 5.69, *p* = 0.02), and depression (*F* (1, 230) = 7.22, *p* = 0.01). In particular, women who experienced a service disruption showed greater levels of general emotional vulnerability and COVID-EMV (see Table 2). They also reported poorer perceived cognitive function, however, the effect was non-significant *(p* = 0.10). There was no main effect of the UK Government shielding letter on COVID-EMV, general anxiety and depression, however, a significant effect was found with perceived cognitive function (*F* (1, 230) = 6.69, *p* = 0.01) with those who received the letter reporting a worse cognitive function (see Table 2). The interaction effect between disruption to scheduled oncology services and UK Government shielding letter was not significant for any of the dependent variables of interest (all *p’*s > 0.05) (see Table 3).

**TABLE 2:** Means and standard deviations for cognitive and emotional health as well as COVID-EMV

	Disruption to oncology services		No disruption to oncology services		Received shielding letter		No shielding letter	
	*M*	SD	*M*	SD	*M*	SD	*M*	SD
Cognitive Ability (FACT-Cog-Total)	82.1	29.8	90.5	28.7	78.1	29.3	90.8	28.7
General Anxiety (HADS-A)	10.4	4.6	9.1	4.4	10.3	5.0	9.3	4.3
General Depression (HADS-D)	7.8	4.4	6.4	3.8	7.7	4.3	6.6	3.9
COVID-Generated Emotional Vulnerability (COVID-EMV)	16.4	6.2	13.7	6.6	15.6	7.2	14.2	6.4
Rumination	49.5	14.2	45.5	14.2	49.5	15.6	45.9	13.8
Pathological Worry (Penn State Worry)	54.6	14.6	50.0	14.8	52.9	15.6	51.0	14.7

**TABLE 3:** ANOVA results using disruption to oncology services and UK Government shielding letter as predictors.

	Sum of Squares	*df*	Mean Square	*F*	*P*
**Anxiety**					
Intercept	15314.11	1	15314.11	774.31	0.00
Disruption to Oncology Services	112.58	1	112.58	5.69	0.02
Government Shielding Letter	27.93	1	27.93	1.41	0.24
Disruption to Oncology Services x Government Shielding Letter	42.26	1	42.26	2.14	0.15
Error	4548.91	230	19.78		
**Depression**					
Intercept	8344.03	1	8344.03	528.22	0.00
Disruption to Oncology Services	113.97	1	113.97	7.22	0.01
Government Shielding Letter	44.08	1	44.08	2.79	0.10
Disruption to Oncology Services x Government Shielding Letter	33.17	1	33.17	2.10	0.15
Error	3633.17	230	15.80		
**COVID-EMV**					
Intercept	36269.65	1	36269.65	863.77	0.00
Disruption to Oncology Services	406.42	1	406.42	9.68	0.00
Government Shielding Letter	57.05	1	57.05	1.36	0.25
Disruption to Oncology Services x Government Shielding Letter	65.3	1	65.30	1.56	0.21
Error	9657.67	230	41.99		
**Cognitive Function**					
Intercept	1086389.00	1	1086389.00	1314.69	0.00
Disruption to Oncology Services	2305.93	1	2305.93	2.79	0.10
Government Shielding Letter	5529.04	1	5529.04	6.69	0.01
Disruption to Oncology Services x Government Shielding Letter	128.20	1	128.20	0.16	0.69
Error	190059.94	230	826.35		

### Impact of Covid-19 Related Distress on Emotional Vulnerability^4^

#### General Anxiety

The results (see Table 4) revealed that the demographic and clinical factors (education, grade, active treatment status, age at diagnosis, time since diagnosis, and health co-morbidity) entered into step one accounted for a small 3.6% of the variance in general anxiety. When measures of rumination and pathological worry were added in step two an additional 52.4% (*R*^2^ change = 0.524; *F* (2, 222) = 132.12, *p* < 0.001) of the variance was explained with both rumination and worry functioning as predictors of general anxiety (*p* < 0.001). On the final step, COVID-EMV predicted significant variance in anxiety with an *R*^2^ (change) of 8.9% (*t* (221) = 7.50, *p* < 0.001), with higher COVID-EMV meeting with higher general anxiety.

**TABLE 4:** Hierarchical regression analyses for the predictors of general anxiety.

	*b*	*SE B*	β	*t*	*p*
**General Anxiety**					
**Step 1**					
Constant	12.15(5.68,18.63)	3.29		3.70	0.00
Education	−0.70(−1.52,0.13)	0.42	–0.11	–1.67	0.10
Grade	0.22(−0.63,1.06)	0.43	0.03	0.50	0.62
Active Treatment Status	1.23(−0.58,3.03)	0.92	0.09	1.34	0.18
Age at Diagnosis	−0.05(−0.13,0.03)	0.04	–0.08	–1.25	0.21
Time since Diagnosis (Months)	−0.02(−0.04,0.00)	0.01	–0.12	–1.76	0.08
Co-morbidity	0.15(−0.49,0.79)	0.32	0.03	0.46	0.65
***Step 2***					
Constant	−1.27(−5.97,3.44)	2.39		–0.53	0.60
Education	−0.43(−0.99,0.13)	0.28	–0.07	–1.50	0.14
Grade	−0.1(−0.68,0.48)	0.29	0.06	–0.35	0.73
Active Treatment Status	0.02(−1.23,1.26)	0.63	0.00	0.02	0.98
Age at Diagnosis	−0.01(−0.07,0.04)	0.03	–0.03	–0.53	0.59
Time since Diagnosis (Months)	0.01(−0.01,0.02)	0.01	0.04	0.84	0.40
Co-morbidity	0.08(−0.36,0.51)	0.22	0.02	0.34	0.74
Rumination	0.13(0.10,0.16)	0.02	0.42	7.60	0.00
Pathological Worry	0.13(0.10,0.16)	0.02	0.44	8.31	0.00
***Step 3***					
Constant	−2.15(−6.37,2.07)	2.41		–1.01	0.32
Education	−0.40(−0.90,0.10)	0.25	–0.06	–1.57	0.12
Grade	−0.11(−0.62,0.41)	0.26	–0.02	–0.40	0.69
Active Treatment Status	0.62(−0.51,1.74)	0.57	0.05	1.08	0.28
Age at Diagnosis	−0.02(−0.07,0.03)	0.02	–0.03	–0.76	0.45
Time since Diagnosis (Months)	0.01(0.00,0.02)	0.01	0.08	1.89	0.06
Co-morbidity	0.16(−0.23,0.55)	0.20	0.03	0.83	0.41
Rumination	0.09(0.06,0.12)	0.02	0.28	5.35	0.00
Pathological Worry	0.08(0.05,0.11)	0.02	0.27	5.24	0.00
COVID-EMV	0.28(0.20,0.35)	0.04	0.41	7.50	0.00

*95% Confidence Intervals.*

#### Depression

The second regression analysis (see Table 5) showed that the model with the six demographic/clinical predictors accounted for a moderate 6.6% of the variance in women’s general depression. Once the predictors worry and rumination were entered step two explained a further 33.5% of the variance (*R*^2^ change = 0.335, *F* (2, 222) = 62.03, *p* < 0.001). Both rumination and worry were significant predictors of depression (*p* < 0.05). In the final step, COVID-EMV added a further 3.1% in explaining general depression after allowing for the other variables (*t* (221) = 3.50, *p* = 0.001). Greater COVID-EMV met with a higher level of depression. Comorbidity was also a significant predictor on the third step.

**TABLE 5:** Hierarchical regression analyses for the predictors of general depression.

	*b*	*SE B*	β	*t*	*p*
**General Depression**					
**Step 1**					
Constant	10.83(5.08,16.59)	2.92		3.71	0.00
Education	−0.42(−1.15,0.31)	0.37	–0.07	–1.13	0.26
Grade	0.65(−0.10,1.40)	0.38	0.11	1.70	0.09
Active Treatment Status	−0.13(−1.73,1.48)	0.81	–0.01	–0.16	0.88
Age at Diagnosis	−0.07(−0.14,0.00)	0.04	–0.12	–1.87	0.06
Time since Diagnosis (Months)	−0.02(−0.04,−0.00)	0.01	–0.17	–2.51	0.01
Co-morbidity	0.65(0.08,1.21)	0.29	0.15	2.24	0.03
**Step 2**					
Constant	2.18(−2.78,7.13)	2.51		0.87	0.39
Education	−0.25(−0.84,0.34)	0.30	–0.04	–0.82	0.41
Grade	0.33(−0.28,0.94)	0.31	0.06	1.07	0.29
Active Treatment Status	−1.23(−2.54,0.08)	0.67	–0.10	–1.85	0.07
Age at Diagnosis	−0.04(−0.10,0.01)	0.03	–0.08	–1.48	0.14
Time since Diagnosis (Months)	−0.00(−0.02,0.01)	0.01	–0.02	–0.32	0.75
Co-morbidity	0.53(0.08,0.99)	0.23	0.12	2.30	0.02
Rumination	0.14(0.10,0.18)	0.02	0.49	7.72	0.00
Pathological Worry	0.05(0.01,0.08)	0.02	0.18	2.84	0.01
**Step 3**					
Constant	1.70(−3.14,6.54)	2.46		0.69	0.49
Education	−0.23(−0.81,0.36)	0.29	–0.04	–0.79	0.43
Grade	0.33(−0.27,0.92)	0.30	0.06	1.09	0.28
Active Treatment Status	−0.91(−2.20,0.38)	0.66	–0.07	–1.39	0.17
Age at Diagnosis	−0.04(−0.10,0.01)	0.03	–0.08	–1.59	0.11
Time since Diagnosis (Months)	0.00(−0.01,0.01)	0.01	0.01	0.13	0.90
Co-morbidity	0.58(0.14,1.03)	0.23	0.14	2.56	0.01
Rumination	0.12(0.08,0.15)	0.02	0.41	6.20	0.00
Pathological Worry	0.02(−0.02,0.06)	0.02	0.08	1.16	0.25
COVID-EMV	0.15(0.07,0.23)	0.04	0.24	3.50	0.00

*95% Confidence Intervals.*

#### Perceived Cognitive Function

The final regression analysis (see Table 6) showed that the demographic/clinical predictors explained 5.4% of the variance in perceived cognitive function. When rumination and worry were then entered in step two an extra 21.9% (*R*^2^ change = 0.219; (*F* (2, 22) = 33.52, *p* < 0.001) of the variance was explained and rumination acted as a significant predictor (*p* < 0.001). On the third step, COVID-EMV predicted significant variance in cognitive function with an *R*^2^ (change) of 3.3% (*t* (221) = -3.25, *p* = 0.001) such that lower perceived cognitive function met a higher level of COVID-EMV. Checks for violation of assumptions using residuals showed that assumptions of collinearity (Tolerance > 0.1, VIF < 10), independent error (General anxiety: Durbin–Watson = 2.00; General depression: Durbin–Watson = 2.12; Perceived cognitive ability: Durbin–Watson = 2.05), normality and homogeneity of variance and linearity were met for anxiety, depression, and cognitive function.

**TABLE 6:** Hierarchical regression analyses for the predictors of perceived cognitive function.

	*b*	*SE B*	β	*t*	*p*
**Perceived Cognitive Function**					
**Step 1**					
Constant	93.04(51.05,135.04)	21.31		4.37	0.00
Education	2.38(−2.96,7.72)	2.71	0.06	0.88	0.38
Grade	−5.66(−11.15,−0.18)	2.78	–0.13	–2.04	0.04
Active Treatment Status	−8.83(−20.55,2.88)	5.95	–0.10	–1.49	0.14
Age at Diagnosis	0.31(−0.20,0.82)	0.26	0.08	1.21	0.23
Time since Diagnosis (Months)	0.10(−0.02,0.22)	0.06	0.11	1.65	0.10
Co-morbidity	−3.87(−8.01,0.27)	2.10	–0.12	–1.84	0.07
**Step 2**					
Constant	138.18(98.62,177.74)	20.08		6.88	0.00
Education	1.46(−3.25,6.17)	2.39	0.04	0.61	0.54
Grade	−3.55(−8.41,1.32)	2.47	–0.08	–1.44	0.15
Active Treatment Status	−1.78(−12.26,8.70)	5.32	–0.02	–0.34	0.74
Age at Diagnosis	0.18(−0.27,0.63)	0.23	0.05	0.80	0.43
Time since Diagnosis (Months)	−0.02(−0.12,0.09)	0.06	–0.02	–0.28	0.78
Co-morbidity	−3.04(−6.69,0.62)	1.86	–0.01	–1.64	0.10
Rumination	−0.95(−1.24,−0.67)	0.14	–0.46	–6.60	0.00
Pathological Worry	−0.09(−0.36,0.17)	0.13	–0.05	–0.70	0.48
**Step 3**					
Constant	141.70(102.91,180.50)	19.69		7.20	0.00
Education	1.35(−3.26,5.97)	2.34	0.03	0.58	0.56
Grade	−3.53(−8.30,1.23)	2.42	–0.08	–1.46	0.15
Active Treatment Status	−4.17(−14.53,6.19)	5.26	–0.05	–0.79	0.43
Age at Diagnosis	0.20(−0.24,0.64)	0.22	0.05	0.89	0.38
Time since Diagnosis (Months)	−0.04(−0.15,0.07)	0.05	–0.04	–0.70	0.48
Co-morbidity	−3.39(−6.98,0.20)	1.82	–0.11	–1.86	0.06
Rumination	−0.78(−1.08,−0.49)	0.15	–0.38	–5.19	0.00
Pathological Worry	0.10(−0.8,0.40)	0.14	0.05	0.73	0.47
COVID-EMV	−1.10(−1.77,−0.43)	0.34	–0.25	–3.25	0.00

*95% Confidence Intervals.*

## Discussion

The current study investigated the impact of the COVID-19 pandemic on cognitive and emotional health in a group of women affected by primary breast cancer. The breast cancer population are at high-risk for developing clinical levels of emotional disorder due to the distressing nature of diagnosis, treatment and long-term side effects (Carreira et al., 2018). The COVID-19 crisis has not only disrupted oncology services but has further required extremely vulnerable groups to follow social shielding guidance for their protection. It follows that such changes may prompt further distress in this population and as such, it is imperative that the effects of COVID-19 related stress are examined. Findings firstly indicate that those who experienced disruptions to their oncology services had higher levels of general anxiety, depression and COVID-19 related emotional vulnerability. Further, results show that those who received a UK Government shielding letter had worse perceived cognitive functioning. This suggests that the indirect consequences of COVID-19 (i.e., oncology service disruption and behavior change requirements) has resulted in worse cognitive and emotional health outcomes in the breast cancer population.

In line with our predictions, further exploration demonstrated that trait worry and rumination were significant predictors of general anxiety and depression respectively. Both rumination and worry are defined by their intrusive and persistent nature and as such have been identified as key cognitive predictors of affective disorder (Nolen-Hoeksema, 2000). Similarly, they have further been associated with deficits in cognitive efficiency (Beckwé et al., 2014; see Koster et al., 2017 for a review). Supporting this, we found that rumination was a significant predictor of worse perceived cognitive function. Of further pertinence, findings indicate that comorbidity with other health conditions significantly predicted higher levels of depression and worse cognitive functioning. This corroborates current reviews that point to the detrimental effects of multimorbidity on psychological health, with studies indicating that depression is two to three times more likely in individuals with multiple health conditions (Read et al., 2017). Finally, of critical importance, after controlling for the predictive value of all demographic variables, multimorbidity as well as worry and rumination, findings demonstrated that COVID-19 related emotional distress was a significant predictor for worse outcomes of anxiety, depression and perceived cognitive function. This suggests that the restrictions relating to the COVID-19 pandemic has emphasized pre-existing levels of cognitive and emotional vulnerability in women affected by primary breast cancer. Pertinently, our analyses controled for potential confounding effects of clinical variables including active treatment status, grade of breast cancer, age of diagnosis, and time since diagnosis. This therefore suggests that findings are applicable to women affected by breast cancer at varying disease and treatment stages, and not only those with more severe cancer-specific vulnerabilities. In the same way, our analyses controlled for the influential effects of trait worry and rumination, which in line with current research, we would typically expect to predict anxiety and depression. Findings indicate that once the predictive value of worry and rumination have been accounted for, COVID-19 related distress was still predictive of generalized anxiety and depression. This suggests that the emotional stress experienced by women affected by breast cancer is over and above pre-existing levels as a result of the restrictions relating to COVID-19. Considering that psychological distress is already established as a common component of the breast cancer experience (Carreira et al., 2018), this findings has important implications. Moreover, emotional distress in women affected by breast cancer has been associated with reduced treatment compliance (Greer et al., 2008), which may influence disease progression and mortality (Satin et al., 2009). Accordingly, any additional distress brought about the COVID-19 pandemic is a noteworthy concern for this population. Furthermore, cancer-related cognitive impairment is a common component of the breast cancer experience (Ahles and Root, 2018), which can greatly impact quality of life. As such, elevated levels of cognitive inefficiency may have considerable consequences for everyday functioning in this population. That said, the above findings must be interpreted with caution. Whilst results show COVID-EMV predicts worse levels of anxiety, depression and cognitive functioning, the relationship may be bidirectional. That is, worse anxiety, depression and cognitive functioning may also predict greater levels of COVID-EMV. Similarly, there may still be a third variable not controlled for in the current analyses, which may have influenced our outcomes.

Overall, the current findings corroborate and add to recent research which suggests that vulnerable groups with pre-existing health conditions, such as those with a breast cancer diagnosis, are likely to suffer worse outcomes as a result of the COVID-19 pandemic (Lai et al., 2020; Zhang et al., 2020). Cancer patients can experience longer term immunosuppressant effects of cancer and its treatment (Kang et al., 2009; Verma et al., 2016), and are further likely to experience multimorbidity with other health conditions (Renzi et al., 2019). As such, the additional indirect effects of treatment delays and disruptions may lead to a significant increase in the psychological burden associated with breast cancer. Further longitudinal research is now required to assess the extent of continued distress associated with COVID-19 disruptions. We advocate that future government preparedness plans consider the mental health implications of pandemics for this population. As such, appropriate eHealth interventions that can be delivered remotely should be further developed and implemented (Penedo et al., 2020). Capitalizing on recent research promoting the effectiveness of online interventions for anxiety and depression in breast cancer may facilitate this aim (Swainston and Derakshan, 2018). As the pandemic progresses, the mental health effects of COVID-19 may be further amplified by longer term effects such as economic recession and continued isolation for vulnerable groups (Gunnell et al., 2020; Holmes et al., 2020). We therefore recommend the continued scientific investigation of COVID-19 related distress in the breast cancer population to identify long-term cognitive and emotional outcomes.

### Limitations

The current study was limited in its scope. As a consequence of shielding and social distancing, participants were recruited via social media platforms and therefore may not be fully representative of the breast cancer population at large. Similarly, our sample lacked diversity; the majority of our participants were caucasian and well-educated. This means that other breast cancer groups, such as those from BAME populations, may be underrepresented in the current study. Emerging data suggests that BAME populations are at increased risk of acquiring the SARS-Cov-2 infection compared to white individuals, and additionally suffer worse clinical outcomes (Pan et al., 2020). As such, we can speculate that increased psychological burden may be even further emphasized in BAME populations. In addition, the UK Government letter categorizing women as vulnerable was sometimes issued at the discretion of the participant’s GP. This means that this group may have had varying degrees of vulnerability. Finally, due to the cross-sectional design of our study, we were unable to measure levels of cognitive and emotional vulnerability pre-pandemic, and as such cannot measure to what extent emotional vulnerability has increased.

## Conclusion

The current study investigated the impact of the COVID-19 outbreak on cognitive and emotional health in breast cancer. Findings suggest that the breast cancer population are at an increased risk of developing psychological disorder during the COVID-19 pandemic. This has critical implications for pandemic preparedness plans, which should consider such consequences. It further highlights the importance of mental health interventions that can be delivered remotely. Future longitudinal research should continue to monitor the longer-term effects of COVID-19 on psychological health in breast cancer.

## Data Availability Statement

The datasets presented in this article are not readily available because the participants of this study did not consent to their data being shared. Requests to access the datasets should be directed to BC, bchapm02@mail.bbk.ac.uk.

## Ethics Statement

The studies involving human participants were reviewed and approved by the Research Ethics Committee of the Department of Psychological Sciences and the College Research Ethics Committee at Birkbeck College, University of London as well as the Economic and Social Research Council. The patients/participants provided their written informed consent to participate in this study.

## Author Contributions

BC, EG, and ND: research question, funding, study design, and analysis plan. BC and JS: preparation of data, data collection, and drafting initial and final version of manuscript. All authors: statistical analysis, critical review of early, and final versions of manuscript.

## Conflict of Interest

The authors declare that the research was conducted in the absence of any commercial or financial relationships that could be construed as a potential conflict of interest.

## Footnotes

1 The BRiC centre (http://briccentre.bbk.ac.uk/) provides an interactive psycho-educational support platform for woman diagnosed with breast cancer in the UK. Currently, the BRiC centre has 1,838 UK members with a diagnosis of breast cancer.

2 Questionnaire available from the authors on request.

3 COVID-19 symptoms were taken from the NHS and Government public support/advise page.

4 Analysis showed that excluding women who reported experiencing a COVID-19 related symptom(s) had no effect on the significance of predictors included in three regression models. Thus, we decided to include all participants in our analyses.
