# Supplementary material for: COVID-19 Lockdown and Its Adverse Impact on Psychological Health in Breast Cancer
Source: Front Psychol. 2020 Aug 24;11:2033. doi: 10.3389/fpsyg.2020.02033 (PMC7476556; doi:10.3389/fpsyg.2020.02033)
Supplement: Supplementary file 1 [file Data_Sheet_1.pdf]

Supplementary Material

Reliability analysis showed that COVID-EMV had excellent reliability, α = .89.

Table 1. Inter-item correlation for COVID-EMV

	COVID-19 Anxiousness	COVID-19 Upset	COVID-19 Fearful	COVID-19 Control	COVID-19 Confidence
COVID-19 Anxiousness	1.00	.79	.62	.72	.63
COVID-19 Upset		1.00	.56	.65	.60
COVID-19 Fearful			1.00	.63	.56
COVID-19 Control				1.00	.62
COVID-19 Confidence					1.00

Table 2. Item-Total Statistics

	Scale Mean if Item Deleted	Scale Variance if Item Deleted	Corrected Item-Total Correlation	Squared Multiple Correlation	Cronbach's Alpha if Item Deleted
COVID-19 Anxiousness	11.16	29.82	.81	.71	.86
COVID-19 Upset	11.56	28.90	.76	.65	.86
COVID-19 Fearful	12.22	28.08	.68	.48	.88
COVID-19 Control	11.37	28.60	.77	.60	.86
COVID-19 Confidence	11.85	27.72	.70	.49	.88

Item-total statistics show that all five items should be retained in COVID-EMV as removal of items would result in a decrease in the Cronbach's α value.

KMO = .87 (Meritorious Value)

Table 3. Factor Analysis for COVID-EMV

	Loading onto Factor One	Communalities
COVID-19 Anxiousness	.89	.78
COVID-19 Upset	.83	.68
COVID-19 Fearful	.82	.52
COVID-19 Control	.74	.68
COVID-19 Confidence	.72	.54
Eigenvalue	3.55	
% of Total Variance	71.05	

Table 4. Correlations between COVID-EMV and cognitive and emotional health questionnaires

	COVID-EMV	HADS-Anxiety	HADS-Depression	Penn State Worry	Rumination Response Scale	FACT-Cog
COVID-EMV	1.00	.70**	.51**	.59**	.56**	-.41**

**Correlation is significant at the 0.01 level
